# Post-recurrence survival analysis in patients with oligo-recurrence after curative esophagectomy

**DOI:** 10.1186/s12885-022-09739-2

**Published:** 2022-06-09

**Authors:** Ping-Chung Tsai, Hung-Che Chien, Po-Kuei Hsu, Jung-Jyh Hung, Chien-Sheng Huang, Wen-Hu Hsu, Han-Shui Hsu

**Affiliations:** 1 Division of Thoracic Surgery, Department of Surgery, Taipei Veterans General Hospital, No. 201, Sec. 2, Shih-Pai Road, Taipei, Taiwan; 2 School of Medicine, National Yang Ming Chiao Tung University, Taipei, Taiwan

**Keywords:** Esophageal cancer, Oligo-recurrence, Post-recurrence survival

## Abstract

**Background:**

Recurrent esophageal cancer is associated with dismal prognosis. There is no consensus about the role of surgical treatments in patients with limited recurrences. This study aimed to evaluate the role of surgical resection in patients with resectable recurrences after curative esophagectomy and to identify their prognostic factors.

**Methods:**

We retrospectively reviewed patients with recurrent esophageal cancer after curative esophagectomy between 2004 and 2017 and included those with oligo-recurrence that was amenable for surgical intent. The prognostic factors of overall survival (OS) and post-recurrence survival (PRS), as well as the survival impact of surgical resection, were analyzed.

**Results:**

Among 654 patients after curative esophagectomies reviewed, 284 (43.4%) had disease recurrences. The recurrences were found resectable in 63 (9.6%) patients, and 30 (4.6%) patients received surgery. The significant prognostic factors of PRS with poor outcome included mediastinum lymph node (LN) recurrence and pathologic T3 stage. In patients with and without surgical resection for recurrence cancer, the 3-year OS rates were 65.6 and 47.6% (*p* = 0.108), while the 3-year PRS rates were 42.9 and 23.5% (*p* = 0.100). In the subgroup analysis, surgery for resectable recurrence, compared with non-surgery, could achieve better PRS for patients without any comorbidities (hazard ratio 0.36, 95% CI: 0.14 to 0.94, *p* = 0.038).

**Conclusions:**

Mediastinum LN recurrence or pathologic T3 was associated with worse OS and PRS in patients with oligo-recurrences after curative esophagectomies. No definite survival benefit was noted in patients undergoing surgery for resectable recurrence, except in those without comorbidities.

## Background

Esophageal cancer is an aggressive gastrointestinal cancer with high rates of recurrence even after curative treatments. Although multimodality treatments combining (neo)adjuvant therapy and radical surgical resection have improved the prognosis for esophageal malignancy [1, 2], postoperative recurrence are still associated with dismal prognosis, with a median survival usually no longer than a year [3, 4]. Around 75% of recurrences occurred in the first 2 years after surgery [5]. Patients were empirically given with systemic therapy with palliative-intent or best supportive care according to the National Comprehensive Cancer Network guidelines, whereas only highly selected patients received potentially curative definitive treatment for limited number of metastases [4].

The concept of oligometastases was first proposed by Hellman in 1995 [6]. It is defined as metastases limited in location and number and perhaps represents tumor early in the chain of progression, not been fully developed and with restricted growth. In theory, it is thought to be more indolent in biological nature and clinical behavior, and may be amenable to a curative therapeutic strategy. Niibe et al. [7] proposed the new notion of oligo-recurrence, similar to oligometastases but with controlled primary lesion. In the state of oligo-recurrence, gross recurrent or metastatic sites could be treated using local therapy. As previous studies [8] have shown, patients with oligo-recurrences had better survival than those with multiple recurrences.

Prior studies [8–10] have shown that selected patients with oligo-recurrent diseases may benefit from aggressive definitive local therapy compared with systemic management alone. However, there is no consensus about the role of surgical resection in these patients. Our purpose is to analyze the post-recurrence survival in patients with resectable recurrences after curative esophagectomy. We aim to identify the prognostic factors and elucidate the role of surgical resection in the management of resectable oligo-recurrence.

## Methods

### Study design and eligibility criteria

We retrospectively reviewed patients receiving esophagectomy for cancer at Taipei Veteran General Hospital between January 2004 and December 2017. To identify patients with resectable recurrences after esophagectomy, we excluded the patients who were (1) at M1 stage, (2) operated on R2 resection, (3) lost from follow-up, (4) without evidence of disease recurrence during follow-up period, and (5) with unresectable recurrences.

The preoperative staging examinations in our protocol included physical examination, laboratory tests, esophagogastroduodenoscopy, flexible bronchoscopy (for upper third and middle third tumors), computed tomography (CT) scans from neck to upper abdomen, and radionuclide bone scans. Endoscopic ultrasound and positron emission tomography/CT (PET/CT) scan became a routine preoperative staging examination for esophageal cancer from 2007. From 2010, multidisciplinary team meetings were held regularly for discussion of examination results and treatment plans. For patients receiving upfront surgery, adjuvant therapy was suggested in patients with pT3/T4 stage and N+ stage. Since the publication of Chemoradiotherapy for Oesophageal Cancer Followed by Surgery Study (CROSS) study, neoadjuvant chemoradiotherapy followed by surgery has become the popular treatment combination for patients with locally advanced tumors. The study protocol was approved by the Institutional Review Board of Taipei Veterans General Hospital and, because of the retrospective nature of the study, informed consent from included patients was waived (approval no. 2015–06-001 BC).

### Follow-up

Clinicopathological stage was determined according to the eighth edition American Joint Committee on Cancer (AJCC) TNM staging system. After curative treatments, all patients were followed routinely every 3 months in the first 2 years, and every 6–12 months after then. Routine follow-up examinations included serum tumor marker, chest radiography, and CT scan from the neck to the upper abdomen. Endoscopy, abdominal sonogram, brain magnetic resonance imaging (MRI), radionuclide bone scans, and PET/CT scan were obtained as clinically indicated.

### Recurrence

Diagnosis of recurrence or metastasis was based on histological, cytological or radiological evidences. Recurrences at the anastomotic site or within the area of previous resection and nodal clearance in the mediastinum or upper abdomen were classified as locoregional recurrence. Distant recurrence was defined as hematogenous metastasis to solid organs or recurrence in the pleura or peritoneal cavity. Resectable lesion was defined as (1) oligo-recurrence, i.e. limited number (≤5) or single site of recurrence, (2) Eastern Cooperative Oncology Group (ECOG) performance status < 2, and (3) surgical approachability based on image studies and determined by two surgeons, PC Tsai and PK Hsu. For example, recurrence in two solid-organ sites or combined failure (simultaneous locoregional and distant recurrences) was considered as disseminated disease that was unresectable. Patient with limited number of lesions in same lobe of liver but judged as not suitable for partial hepatectomy by a multidisciplinary team meeting was defined as unresectable.

### Statistical analyses

Chi-square test was used to compare between categorical variables and independent t test was used to compare between continuous variables. In the survival analysis, overall survival (OS) was defined as the period of time from the curative esophagectomy to death or the last follow-up. Disease free interval (DFI) was defined as the period of time from the curative esophagectomy to the detection of recurrence. The duration between the detection of initial recurrence and either death or the last follow-up was defined as post-recurrence survival (PRS). Survival curves were plotted by Kaplan-Meier method and compared by log-rank test. Cox’s proportional hazards model was used for univariate and multivariate survival analysis. A *p* < 0.05 was defined as indicative of statistical significance. All calculations were performed using IBM SPSS 25.0 software, and picture design was finished based on R version 4.1.1 (The R Foundation for Statistical Computing, Vanderbilt University, Nashville, TN, USA) using the Survival, ggplot2, survminer packages.

## Results

### Patient characteristics

**Fig. 1:**
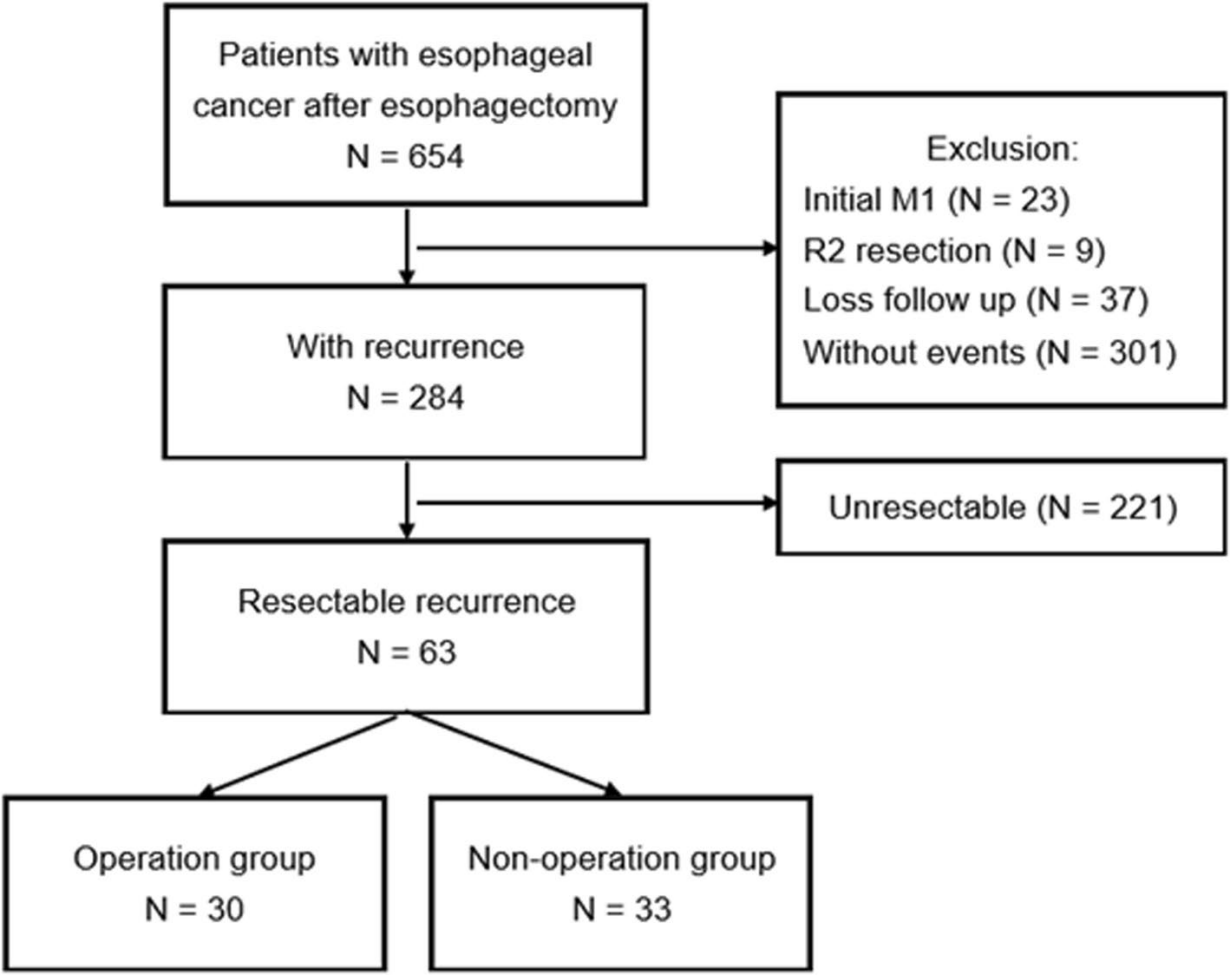
Summary of patient flow chart

A total of 654 patients who underwent esophagectomy between 2004 and 2017 were included, and 284 of them (43.4%) had disease recurrence. After exclusionary screening, 63 patients were deemed with oligo-recurrence amenable for surgical intent. Based on definitive treatments for “resectable” recurrences, these patients were divided into operation group (*n* = 30) and non-operation group (*n* = 33) (Fig. 1). Table 1 shows their clinical and pathologic features. Both groups of patients were overwhelmingly male (96.8%), and had squamous cell carcinoma (95.2%) as the histologic cell type of esophageal cancer. The patients in non-operation group were older (median age: 60 vs. 52 yrs., *p* = 0.003), and had more with locoregional oligo-recurrence (31.7% vs. 11.2%, *p* = 0.003). Otherwise, we found no substantial differences between both groups in parameters such as gender, performance status, Charlson Comorbidity Index (CCI), tumor characteristics, or initial treatment modality of the primary esophageal cancer.

**Table 1:** Clinicopathologic characteristics of patients with resectable recurrent esophageal cancer

	Operation group (*N* = 30)	Non-operation group (*N* = 33)	*p* value
Age, years, median (IQR)	52 (47–59)	60 (53–68)	0.003
Sex (%)			0.945
Male	29 (96.7%)	32 (97.0%)	
Female	1 (3.3%)	1 (3.0%)	
Tumor location (%)			0.285
Upper/middle third	18 (60.0%)	24 (72.7%)	
Lower third/GE junction	12 (40.0%)	9 (27.3%)	
Histologic cell type (%)			0.612
Squamous cell carcinoma	29 (96.7%)	31 (93.9%)	
Adenocarcinoma or other	1 (3.3%)	2 (6.1%)	
Tumor length, cm, median (IQR)	3.5 (2.3–5.0)	3 (2.0–5.0)	0.560
Pathologic T stage (%)			0.222
ypT0	8 (26.7%)	4 (12.1%)	
(y)pT1	6 (20.0%)	6 (18.2%)	
(y)pT2	6 (20.0%)	4 (12.1%)	
(y)pT3	10 (33.3%)	19 (57.6%)	
Pathologic N stage (%)			0.904
N0	15 (50.0%)	17 (51.5%)	
N1/N2/N3	15 (50.0%)	16 (48.5%)	
Differentiation (%)			0.334
Grade X (cannot be assessed)	8 (26.7%)	5 (15.2%)	
Well/moderately	18 (60.0%)	21 (63.6%)	
Poorly	4 (13.3%)	7 (21.2%)	
Lymphovascular invasion (%)			0.961
Negative	18 (60.0%)	20 (60.6%)	
Positive	12 (40.0%)	13 (39.4%)	
Perineural invasion (%)			0.540
Negative	27 (90.0%)	28 (84.8%)	
Positive	3 (10.0%)	5 (15.2%)	
Circumferential margin (%)			0.224
Uninvolved	27 (90.0%)	26 (78.8%)	
Involved/close	3 (10.0%)	7 (21.2%)	
Treatment modality (%)			0.108
Neoadjuvant treatments + surgery	13 (43.3%)	8 (24.2%)	
Upfront surgery	17 (56.7%)	25 (75.8%)	
Initial surgery method			0.476
Mckeown esophagectomy	29 (96.7%)	33 (100.0%)	
Ivor lewis approach	1 (3.3%)	0	
Tumor regression grade (%)			0.948
0	8 (26.7%)	4 (12.1%)	
1	3 (10.0%)	2 (6.1%)	
2	1 (3.3%)	1 (3.0%)	
3	1 (3.3%)	1 (3.0%)	
Disease free interval (%)			
≤ 12 months	13 (43.3%)	18 (54.5%)	0.374
> 12 months	17 (56.7%)	15 (45.5%)	
Charlson Comorbidity Index (%)			0.547
0	15 (50.0%)	19 (57.6%)	
≥ 1	15 (50.0%)	14 (42.4%)	
Performance status (%)			0.409
0	22 (73.3%)	21 (63.6%)	
1	8 (26.7%)	12 (36.4%)	
Recurrence pattern (%)			0.003
Locoregional recurrence	7 (23.3%)	20 (60.6%)	
Distant metastasis	23 (76.7%)	13 (39.4%)	
Total recurrence (%)			0.002
Anastomosis	1 (3.3%)	4 (12.1%)	
Cervical lymph node	5 (16.7%)	6 (18.2%)	
Mediastinal lymph node	1 (3.3%)	10 (30.3%)	
Lung	17 (56.7%)	4 (12.1%)	
Chest wall/ pleura seeding	2 (6.7%)	3 (9.1%)	
Liver	1 (3.3%)	3 (9.1%)	
Brain	3 (10.0%)	0	
Bone	0	2 (6.1%)	
Spleen	0	1 (3.0%)	

*IQR* Interquartile range

### Recurrence pattern and treatment modalities

During the mean follow-up of 38 months, the median period of time to develop recurrence was 13 months. The patterns of recurrences were distant only in 36 (36/63, 57.1%) patients, and locoregional only in 27 (27/63, 42.9%) patients (Table 1). The treatment modalities for resectable recurrences are shown in Table 2. In the operation group, surgical resection followed by chemotherapy (53.3%) was the most common treatment combination. Most (80%) of the patients had additional treatments after surgery, whereas the other 20% had surgery only. In the non-operation group, chemoradiotherapy (42.4%) was the most common treatments, followed by chemotherapy only (27.3%) and local radiotherapy only (12.1%). Specifically in the operation group (among 63 patients having resectable recurrences), one of five patients with anastomosis recurrence underwent revision of esophagogastric anastomosis; 17 of 21 patients with isolated lung metastasis had lung resection. One of four patients with liver oligo-recurrence received lateral hepatectomy and 3 other patients had radiofrequency ablation. One patient with pleural/chest wall oligo-recurrence underwent cryoablation plus radiotherapy.

**Table 2:** Treatment modalities of for resectable recurrent esophageal cancer

Modalities of treatments	Number
Operation group	30
Surgery with chemoradiotherapy	6 (20%)
Surgery with chemotherapy	16 (53.3%)
Surgery with radiotherapy	2 (6.7%)
Surgery Only	6 (20%)
Non-operation group	33
Chemoradiotherapy	14 (42.4%)
Chemotherapy	9 (27.3%)
Local radiotherapy	4 (12.1%)
Radiofrequency ablation	3 (9.1%)
Cryoablation + radiotherapy	1 (3.0%)
Palliative care	2 (6.1%)

### Factors of PRS and OS

The overall 1- and 3-year PRS rates were 62.7 and 31.6%. Median survival for all patients after recurrence was 18 months (interquartile range (IQR): 8 to 64 months). The prognostic factors of PRS are shown in Table 3. In the multivariate analysis with forward selection criteria, pathologic T3 stage (hazard ratio 2.53, 95% CI: 1.36 to 4.71, *p* = 0.003) and mediastinum recurrence (hazard ratio 3.81, 95% CI: 1.76 to 8.26, *p* = 0.006) were the independent prognostic factors of PRS.

**Table 3:** Prognostic factors of post-recurrence survival

Variable	Univariate Analysis		Multivariate Analysis	
	HR (95% CI)	*p* value	OR (95% CI)	*p* value
Age (≥ 60 vs. < 60 yrs)	1.42 (0.77–2.61)	0.259		
Sex (male vs. female)	0.05 (0.01–22.32)	0.329		
Charlson Comorbidity Index (≥1 vs. 0)	1.18 (0.66–2.11)	0.575		
Performance status (1 vs. 0)	1.25 (0.68–2.32)	0.475		
Tumor location (U/M third vs. L third/EGJ)	1.26 (0.68–2.32)	0.457		
Tumor length	0.97 (0.82 ~ 1.14)	0.697		
Neoadjuvant therapy (yes vs. no)	0.96 (0.51–1.78)	0.885		
(y)p T stage (T3 vs. T0–2)	2.03 (1.13–3.65)	0.018	2.53 (1.36–4.71)	0.003
(y)p N stage (N+ vs. N0)	1.39 (0.77–2.49)	0.270		
Differentiation (poorly vs. well + moderately)	1.88 (0.78–4.53)	0.161		
LVI (+ vs. -)	1.32 (0.74–2.36)	0.353		
PNI (+ vs. -)	1.71 (0.76–3.86)	0.197		
TRG (0 vs. 1–3)	0.59 (0.24–1.41)	0.244		
CRM (involved or close vs. uninvolved)	1.34 (0.63 ~ 2.84)	0.440		
Disease free interval (> 12 vs. ≤ 12 months)	0.65 (0.36–1.17)	0.149		
Recurrence pattern				
Distant vs. loco-regional	0.81 (0.45–1.45)	0.475		
Lung (with vs. without)	0.60 (0.31–1.15)	0.125		
Liver (with vs. without)	1.02 (0.32–03.32)	0.967		
Bone (with vs. without)	1.89 (0.450–7.88)	0.382		
Brain (with vs. without)	2.69 (0.81–8.88)	0.105		
Pleural/Chest wall (with vs. without)	0.95 (0.33–2.69)	0.923		
Cervical lymph node (with vs. without)	0.85 (0.39–1.83)	0.676		
Mediastinum (with vs. without)	2.79 (1.35–05.75)	0.005	3.81 (1.76–8.26)	0.001
Anastomosis (with vs. without)	0.41 (0.10–1.78)	0.236		
Operation for recurrence (yes vs. no)	0.61 (0.34 ~ 1.11)	0.107		

*HR* Hazard ratio, *CI* Confidence interval, *U* Upper, *M* Middle, *L* Lower, *EGJ* Esophagogastric junction, *LVI* Lymphovascular invasion, *PNI* Perineural invasion, *TRG* Tumor regression grade, *CRM* Circumferential resection margin

In the univariate analysis (Table 4), pathologic T3 stage, DFI less than 12 months, brain metastasis, and mediastinal recurrence demonstrated to be significant factors of OS. Similar to the findings of PRS, pathologic T3 stage (hazard ratio 3.17, 95% CI: 1.57 to 6.37, *p* = 0.001) and mediastinum recurrence (hazard ratio 4.21, 95% CI: 1.88 to 9.43, *p* = 0.001) were also the poor prognostic factors of OS. Additionally, we found brain metastasis (hazard ratio 8.21, 95% CI: 2.19 to 30.78, *p* = 0.002) as an independent prognostic factor of worse OS.

**Table 4:** Prognostic factors of overall survival

Variable	Univariate Analysis		Multivariate Analysis	
	HR (95% CI)	*p* value	OR (95% CI)	*p* value
Age (≥60 vs. < 60 yrs)	1.42(0.77 ~ 2.60)	0.256		
Sex (male vs. female)	0.05(0.01 ~ 18.45)	0.314		
Charlson comorbidity index (≥1 vs. 0)	1.46(0.78 ~ 2.71)	0.232		
Performance status (1 vs. 0)	1.12(0.63 ~ 2.01)	0.691		
Tumor location (U/M third vs. L third/EGJ)	1.12(0.61 ~ 2.05)	0.717		
Tumor length	1.01(0.86 ~ 1.18)	0.882		
Neoadjuvant therapy (yes vs. no)	0.93(0.50 ~ 1.73)	0.820		
(y)p T stage (T3 vs. T0–2)	2.64(1.46 ~ 4.75)	0.001	3.17(1.57 ~ 6.37)	0.001
(y)p N stage (N+ vs. N0)	1.59(0.89 ~ 2.85)	0.116		
Differentiation (poorly vs. well + moderately)	2.07(0.86 ~ 4.97)	0.105		
LVI (+ vs. -)	1.61(0.90 ~ 2.89)	0.107		
PNI (+ vs. -)	1.33(0.59 ~ 3.01)	0.486		
TRG (0 vs. 1–3)	0.55(0.23 ~ 1.31)	0.176		
CRM (involved or close vs. uninvolved)	1.92(0.91 ~ 4.03)	0.085		
Disease free interval (> 12 vs. ≤ 12 months)	0.39(0.22 ~ 0.71)	0.002	0.54(0.28 ~ 1.03)	0.062
Recurrence pattern				
Distant vs. loco-regional	0.87(0.48 ~ 1.56)	0.638		
Lung (with vs. without)	0.57(0.30 ~ 1.08)	0.086		
Liver (with vs. without)	1.03(0.32 ~ 3.35)	0.956		
Bone (with vs. without)	3.19(0.76 ~ 13.46)	0.114		
Brain (with vs. without)	3.83(1.13 ~ 12.94)	0.031	8.21(2.19 ~ 30.78)	0.002
Pleural/Chest wall (with vs. without)	1.17(0.42 ~ 3.30)	0.760		
Cervical lymph node (with vs. without)	1.01(0.47 ~ 2.17)	0.972		
Mediastinum (with vs. without)	2.37(1.15 ~ 4.85)	0.019	4.21(1.88 ~ 9.43)	0.001
Anastomosis (with vs. without)	0.26(0.60 ~ 1.15)	0.077		
Operation for recurrence (yes vs. no)	0.62(0.34 ~ 1.12)	0.114		

*HR* Hazard ratio, *CI* Confidence interval, *U* Upper, *M* Middle, *L* Lower, *EGJ* Esophagogastric junction, *LVI* Lymphovascular invasion, *PNI* Perineural invasion, *TRG* Tumor regression grade, *CRM* Circumferential resection margin

### Impact of treatment modality on survival

**Fig. 2:**
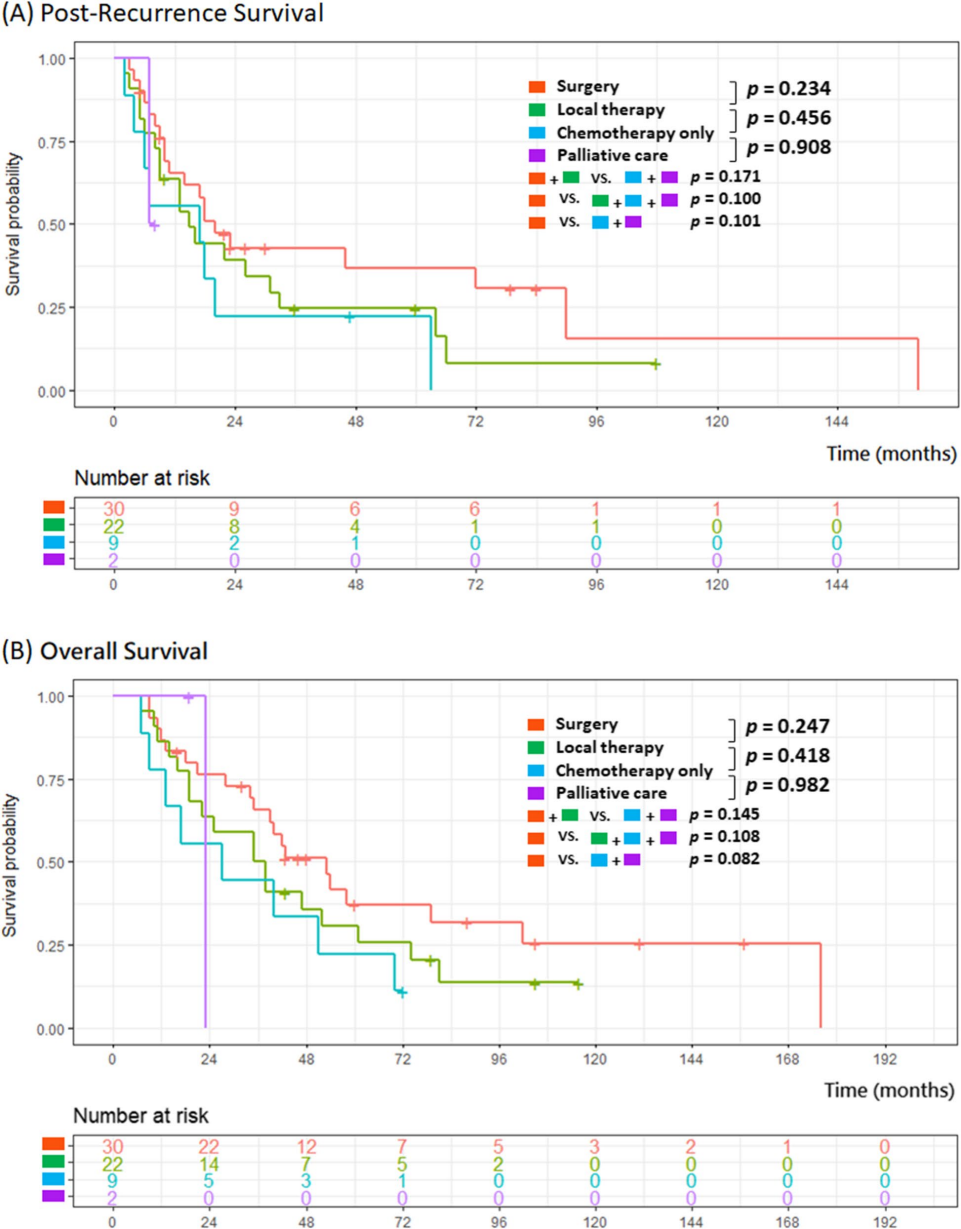
Post-recurrence survival curve (**A**) and Overall survival curve (**B**) for the modalities of treatment

**Fig. 3:**
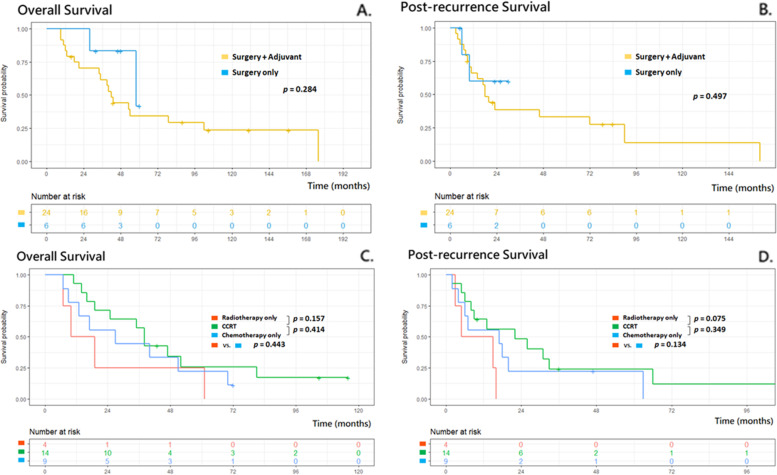
Survival curves of patients in operation group (**A**, **B**) and non-operation group (**C**, **D**) under different treatments

Figure 2 demonstrates the impact curves of different treatment modalities on patient outcome (A: PRS; B: OS). Patients were divided into surgery (surgical resection +/− systemic therapy), local therapy (radiation therapy/radiofrequency ablation/cryoablation +/− systemic therapy), chemotherapy only, and palliative care groups. Although the patients who underwent surgery seemed to have better outcomes--median OS: 42.5 months, interquartile range (IQR):20.3–64.8; and median PRS: 18 months, no statistical significance was reached. There was no significant difference between surgery and non-surgery groups (median OS: 35 months (IQR:16.5–56.5); median PRS: 13 months; *p* = 0.108 in PRS and *p* = 0.100 in OS), and between any types of local treatments and systemic treatments (median OS: 39.5 months (IQR:19–60.8) vs. 23 (IQR:13–51), *p* = 0.171; median PRS: 16.5 vs 8 months, *p* = 0.145). Further subgroup analysis of patient who underwent surgery (with adjuvant therapy or not) or alternative treatment (chemo-radiotherapy/chemotherapy only/ radiotherapy only) were demonstrated in Fig. 3, with limited sample size in some groups. No significant difference between surgery alone and surgery with adjuvant therapy groups (median OS: 41 (IQR:22.8–57) vs. 42.5 months (IQR:22.8–59.5), *p* = 0.284; median PRS: 17.5 (IQR:9–25.3) vs 18 months (IQR:9–29), *p* = 0.497). Between chemo-radiotherapy /chemotherapy only/ radiotherapy only treatment groups (median OS: 45.5 vs. 31 vs 31 months; median PRS: 13 vs 15.5 vs 17 months, respectively) were no statistical significance in each group comparison.

**Fig. 4:**
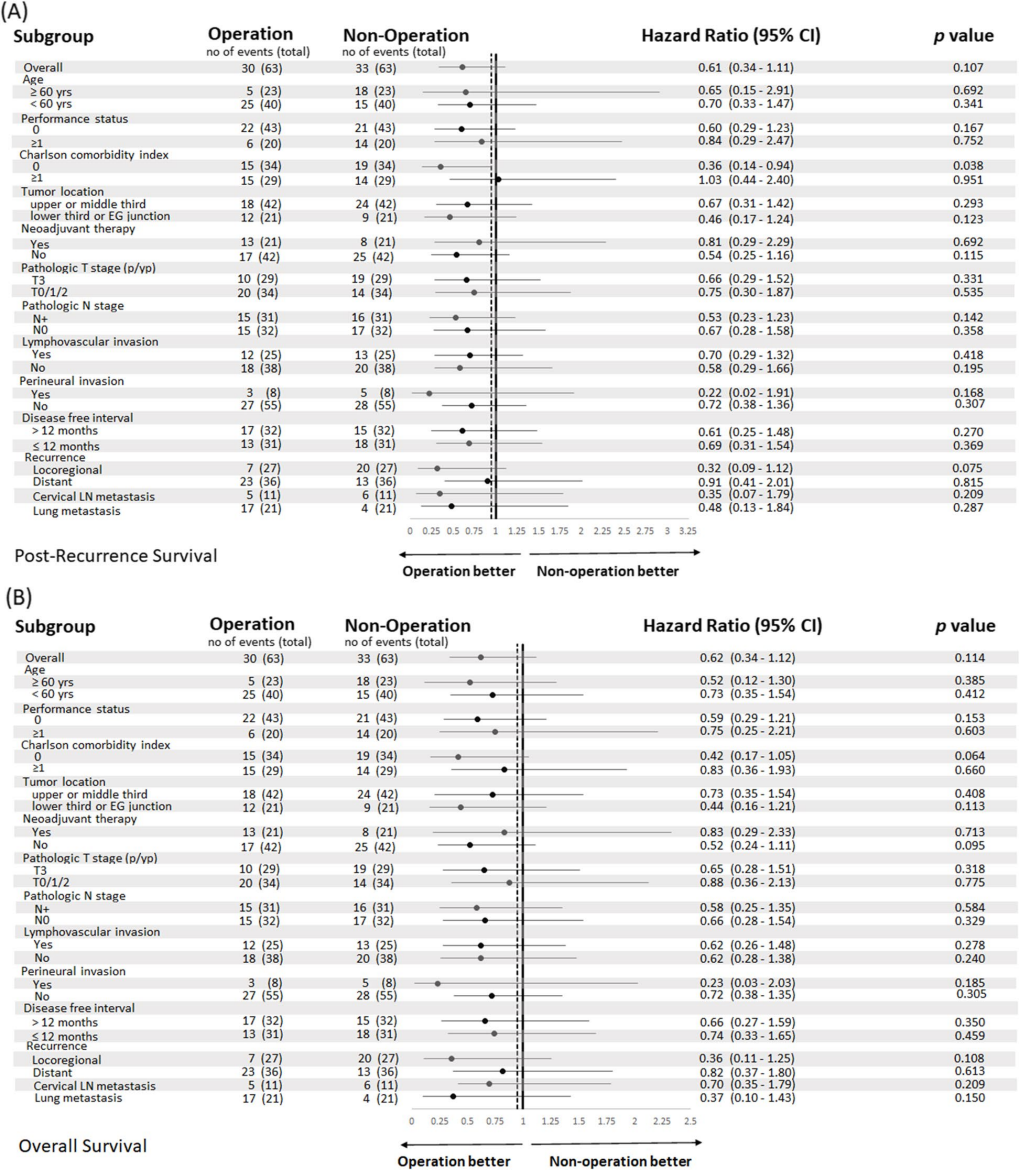
Post-recurrence survival forest plot (**A**) and Overall survival forest plot (**B**) for comparison of operation group and non-operation group

To identify who might benefit from surgical resection for resectable recurrence, survival analysis was undertaken to compare between operation and non-operation groups (Fig. 4). Although operation, in general, led to better outcomes, CCI = 0 was identified as the only indicator to make significant difference between operation and non-operation. Patients without comorbidities could expect significantly better PRS outcome from surgery for resectable recurrence than from non-operation treatments (hazard ratio 0.36, 95% CI: 0.14 to 0.94, *p* = 0.038).

## Discussion

Esophageal cancer is one of the deadliest cancers with rapidly rising incidence. In our previous report [3], tumor recurrence after curative esophagectomy developed in 42.9% of patients in the median of only 10 months. As high as 66.2% of recurrences happened within 1 year after operation. The reported prognostic factors of PRS included liver recurrence, shorter disease-free interval and palliative therapy. Distant recurrence and more than 3 recurrent locations were associated with worse PRS, as demonstrated in K. Parry et al’s study [4]. In current study on patients with oligo-recurrence after esophagectomy, we identified pathological T3 stage and mediastinal recurrence as the prognostic factors of PRS.

In general, patients with multiple recurrent sites had a worse survival compared with those with less involved sites [8]. Combined failure pattern of simultaneous locoregional and distant recurrences also had inferior outcome [9]. Indeed, patients with recurrent esophageal disease deserved multimodality therapy for better outcome. Furthermore, based on the rising publications showing evidence that oligo-recurrence may represent tumors with more favorable biology, early identification and aggressive treatment for oligometastatic recurrence might improve survival [8–12]. However, the role of surgery in patients with isolated oligo-recurrence has not yet been defined.

In Ghaly et al’s study [11], no pronounced difference was found in disease-free survival or in PRS between patients with oligo-recurrence treated with operation, with or without chemo- and radiotherapy, and patients who received definitive chemoradiotherapy without resection. In contrast, the study by Depypere et al. [9] demonstrated prolonged survival in patients with isolated locoregional recurrence or single solid organ metastasis, especially if surgery was offered. Surgical resection (+/− systemic therapy) of solitary recurrent lesion was a considerable therapeutic option for well-selected patients with recurrent esophageal carcinoma. Similarily, Ohkura et al. [8] reported a significantly better OS rate in the patients who underwent resection of oligo-recurrences than in those who did not. However, they found no significant difference in survival between the patients with shorter and longer DFI (< 12 months vs. > 12 months), indicating that a shorter DFI should not be an unfavorable prognostic factor to hinder surgeons from choosing surgical resection. In line with these reports, we observed that surgical resection for oligo-recurrence may lead to better OS and PRS, albeit without statistical significance. Most of our patient (95.2%) histologically were classified as squamous cell carcinoma, which has been well demonstrated more chemo-radiosensitive and better pathologic response than adenocarcinoma [2], may have influenced our analysis. However, in subgroup analysis, we found that surgery, compared with non-surgical treatments, may confer survival benefit to patients without comorbidities, highlighting the importance of patient selection in deciding treatment modalities. The cause of death after recurrence might be multi-faceted and complex, patients without comorbidities may well tolerate both surgery and subsequent therapy side effect side-effects without fatal toxicity in our study.

Cervical and mediastinal lymph node failures have been reported to be one of the most common types of recurrence after esophagectomy in patients with thoracic esophageal squamous cell carcinoma [3, 13]. In Ni’s study [13], lymph node recurrence above the diaphragm and single region lymph node recurrence exhibited better OS than those at the subphrenic region and multiple regions, respectively. They also identified original pathological stage and salvage treatment regimen as independent prognostic factors. Whereas chemoradiotherapy could offer a safe and effective treatment for patients with lymph node recurrences, especially with a single region failure [14, 15], many reports suggested that salvage cervical lymphadenectomy as the main treatment could achieve locoregional disease control and prolong survival in patients with cervical lymph node (LN) recurrence after curative esophagectomy [16]. Focusing on lymph node recurrence, Nakamura et al. have shown significantly better survival in the lymphadenectomy and chemoradiotherapy groups than in the patients who received chemotherapy or best supportive care for lymph node recurrence after curative esophagectomy [17]. However, there was no statistically significant difference in survival between the surgical lymphadenectomy and chemoradiotherapy groups. Of note, 11 of 12 patients with cervical lymph node recurrence received surgical resection, whereas less than one third of patients with paraesophageal or paratracheal lymph node recurrences had surgical resection. These results were compatible with our findings that only one of 11 patients with isolated mediastinal lymph node recurrence received surgery. Mediastinal lymph node recurrence was also a significant prognostic factor of both OS and PRS. Therefore, the possibility and benefit of salvage surgical resection for mediastinal LN recurrence remains unclear and needs more data to clarify.

Limited reports on oligo-recurrence to distant solid organ have made it difficult to collect sufficient cases for analysis. The role of surgery in these patients thus remains unknown. For example, only 30% of the patients with oligo-recurrence had surgery in Nobel’s study [12]. However, several reports have recommended pulmonary metastasectomy as an acceptable and effective treatment for solitary pulmonary metastasis [18–21]. For example, Kobayashi et al. analyzed 23 patients who underwent 30 curative pulmonary metastasectomies at a single institution [18]. In their report, the unfavorable prognostic factors included history of extrapulmonary metastases before pulmonary metastasectomy, poorly differentiated primary esophageal carcinoma, and short disease-free interval. Their results also recommended that pulmonary resection for lung metastases from esophageal carcinoma should be considered in carefully selected patients, and repeated metastasectomy was encouraged in suitable patients.

On the other hand, Nobel et al. have reported significantly worse outcome in patients with liver and brain oligo-recurrence from esophageal cancer when compared with lung oligo-recurrence, which showed a more indolent course [12]. In our series, 17 of 21 patients had received pulmonary resection but showed no significant differences in survival analysis compared with patients with oligo-recurrences in other sites. On the contrary, patients with brain oligo-recurrence had worst OS (HR: 3.83, *p* = 0.031) compared with patients with oligo-recurrences in other sites, which are compatible with the findings in Nobel’s cohort. Whether patients with esophageal cancer should be screened or surveyed for brain metastases remains unclear [22–24]. Although a previous study [22] has reported that half of brain oligo-recurrences occurred within 12 months of esophagectomy and all were diagnosed because of symptomatic disease, which led to the suggestion of brain surveillance imaging for high risk patients, there was report showing low incidence of brain metastasis in patients with esophageal carcinoma, which made it unnecessary for baseline screening or surveillance, even the prognosis was poor [23].

There are several limitations in our study. First, this is a single-institution study. The inherent bias of retrospective nature and relatively small sample size may limit the power of statistical significance. Second, due to the lack of strict definition for “resectable” and guidelines for therapeutic approach in each recurrence site, selection bias for treatment modalities and surgical intervention are inevitable. Third, routine surveillance for bone and brain was not performed in our practice, which might miss early diagnosis of oligo-recurrences in these sites. Finally, since only patients with single site of recurrence were selected, the role of surgery in combination with other aggressive local control or systemic therapy in patients with limited or multiple sites of recurrence was beyond the scope of our study. External validation study with more cases is needed to confirm our observations.

## Conclusions

Patients with oligo-recurrence represent a small subgroup of patients with recurrence, surgical resection may offer a survival benefit for properly selected patients. We demonstrated that patients with mediastinum LN recurrence or pathologic T3/4 stage were independent factors of OS and PRS for those who had oligo-recurrences after esophagectomy. In patients without comorbidities (CCI = 0), surgical resection was associated with better PRS. Further studies are warranted to define the role of surgical resection in the management of resectable oligo-recurrence.

## Data Availability

The datasets during and/or analyzed during the current study available from the corresponding author on reasonable request and IRB approval.

## Abbreviations

OS: Overall survival
PRS: Post-recurrence survival
DFI: Disease free interval
CT: Computed tomography
PET: Positron emission tomography
MRI: Magnetic resonance imaging
CROSS: Chemoradiotherapy for Oesophageal Cancer Followed by Surgery Study
AJCC: American Joint Committee on Cancer
ECOG: Eastern Cooperative Oncology Group
CCI: Charlson Comorbidity Index
EGJ: Esophagogastric junction
LVI: Lymphovascular invasion
PNI: Perineural invasion
TRG: Tumor regression grade
CRM: Circumferential resection margin
LN: Lymph node
